# Genome of the Rio Pearlfish (*Nematolebias whitei*), a bi-annual killifish model for Eco-Evo-Devo in extreme environments

**DOI:** 10.1093/g3journal/jkac045

**Published:** 2022-02-21

**Authors:** Andrew W Thompson, Harrison Wojtas, Myles Davoll, Ingo Braasch

**Affiliations:** 1 Department of Integrative Biology, Michigan State University, East Lansing, MI 48824, USA; 2 Ecology, Evolution & Behavior (EEB) Program, Michigan State University, East Lansing, MI 48824, USA; 3 Department of Biology, University of Virginia, Charlottesville, VA 22903, USA

**Keywords:** *Nematolebias whitei*, Rio Pearlfish, diapause, aging, hatching, extreme environments, Eco-Evo-Devo, teleost

## Abstract

The Rio Pearlfish, *Nematolebias whitei*, is a bi-annual killifish species inhabiting seasonal pools in the Rio de Janeiro region of Brazil that dry twice per year. Embryos enter dormant diapause stages in the soil, waiting for the inundation of the habitat which triggers hatching and commencement of a new life cycle. Rio Pearlfish represents a convergent, independent origin of annualism from other emerging killifish model species. While some transcriptomic datasets are available for Rio Pearlfish, thus far, a sequenced genome has been unavailable. Here, we present a high quality, 1.2 Gb chromosome-level genome assembly, genome annotations, and a comparative genomic investigation of the Rio Pearlfish as representative of a vertebrate clade that evolved environmentally cued hatching. We show conservation of 3D genome structure across teleost fish evolution, developmental stages, tissues, and cell types. Our analysis of mobile DNA shows that Rio Pearlfish, like other annual killifishes, possesses an expanded transposable element profile with implications for rapid aging and adaptation to harsh conditions. We use the Rio Pearlfish genome to identify its hatching enzyme gene repertoire and the location of the hatching gland, a key first step in understanding the developmental genetic control of hatching. The Rio Pearlfish genome expands the comparative genomic toolkit available to study convergent origins of seasonal life histories, diapause, and rapid aging phenotypes. We present the first set of genomic resources for this emerging model organism, critical for future functional genetic, and multiomic explorations of “Eco-Evo-Devo” phenotypes of resilience and adaptation to extreme environments.

## Introduction

Aplocheiloid killifishes inhabit tropical freshwater habitats around the world. Some African and Neotropical species live in ephemeral waters that are subject to seasonal desiccation (Myers 1952; Simpson 1979). Desiccation kills the adults, but embryos survive inside specialized eggs (Thompson *et al.* 2017a) buried in the soil via 3 diapause stages (DI, DII, DIII; Wourms 1972a,1972b, 1972c). DI occurs as a migratory dispersion of blastomeres, DII occurs during somitogenesis when organs are rudimentary, and DIII occurs after organogenesis when the embryo is fully formed and poised to hatch upon habitat inundation. This seasonal life history is a remarkable example of convergent evolution (Furness *et al*. 2015) with 7 gains across killifish evolution (Thompson *et al.* 2021a). Additionally, annual killifishes show rapid aging due to relaxed selection on lifespan (Cui *et al.* 2019) and are an important emerging model system for the study of senescence (Valenzano *et al.* 2011, 2015; Harel *et al.* 2015; Reichwald *et al.* 2015).

**Fig. 1.**
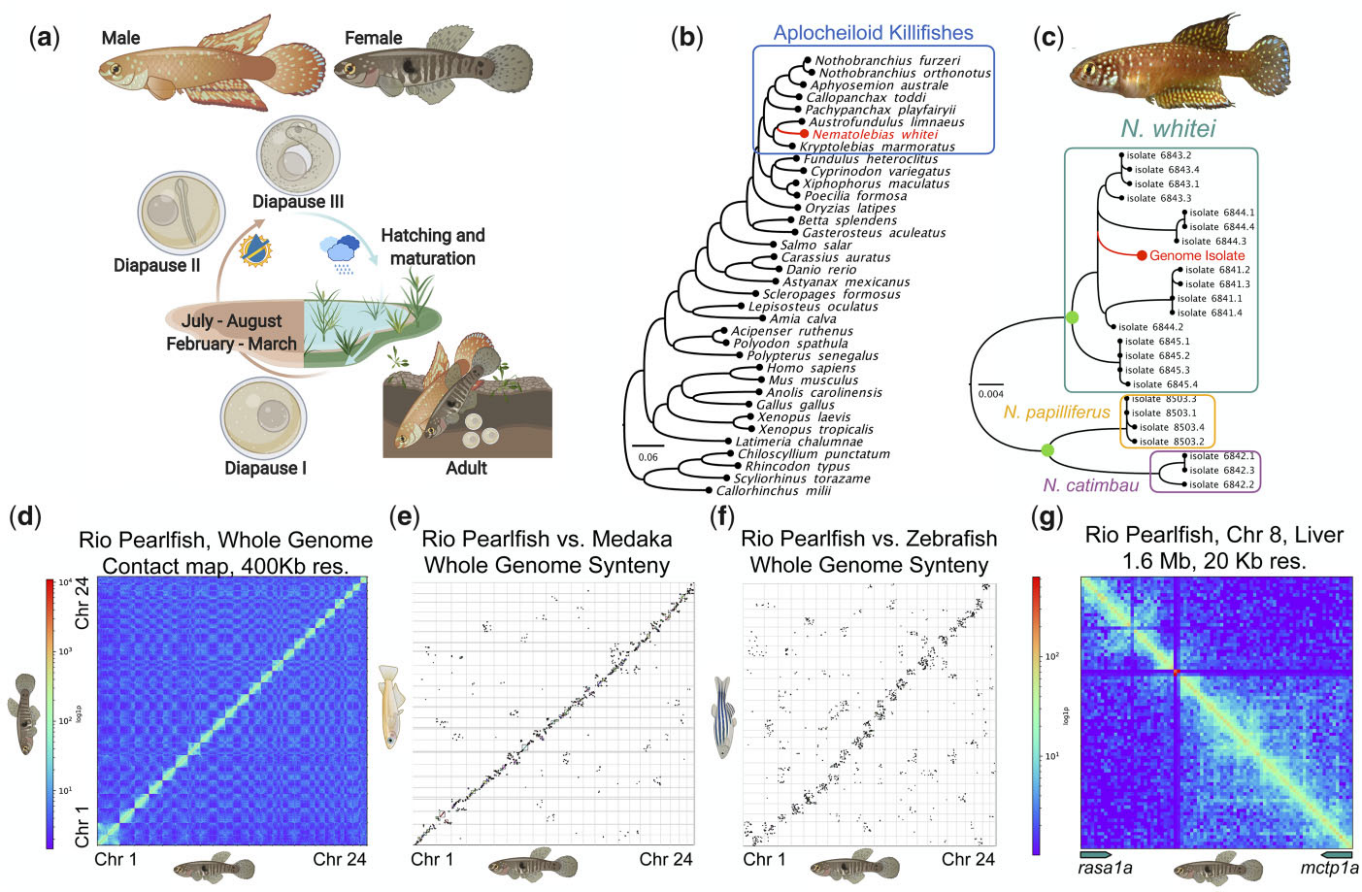
Rio Pearlfish evolution, ecology, development, and 3D genome structure. a) Bi-annual life cycle of the Rio Pearlfish with three developmental diapause stages following burying of eggs in soil. b) Relative position of Rio Pearlfish in the vertebrate tree of life inferred by Orthofinder based on annotated proteins. c) DNA barcode (*cox1* and *cytb*) phylogeny inferred with RAxML of the genus *Nematolebias* confirming the identity of the genome specimen as *N. whitei*. Sequences from Costa *et al.* (2014) were used for comparison to the reference genome sequence. Green nodes show 100% bootstrap support for the reciprocal monophyly of *N. whitei* with other genera and confirms the identity of the genome specimen with high confidence. d) Hi-C contact map of the Rio Pearlfish genome showing linkage of the 24 chromosomes into chromosomal pseudomolecules. e, f) SynMap genome-wide synteny plots of Rio Pearlfish *vs.* medaka (e) and *vs.* zebrafish (f) showing genome-structure conservation across over 250 million years of teleost evolution. g) Hi-C contact maps of the syntenic region between *rasa1a* and *mctp1a* in Pearlfish liver tissue. These contact maps highlight the conserved 3D structure that include TADs conserved across teleost evolution as well as cell types and developmental stages (Nakamura *et al.* 2021). Species graphics Created with BioRender.com.

The Rio Pearlfish, *Nematolebias whitei*, is a seasonal killifish endemic to the coastal plains of the Rio de Janeiro region in Brazil, inhabiting pools that dry twice annually, from July–August and February–March (Fig. 1a;Myers 1942; Costa 2002). Pearlfish represents a separate origin of seasonality from other killifish model species, i.e. *Nothobranchius furzeri* and *Austrofundulus limnaeus* (Thompson *et al.* 2021a). In *N. whitei*, DI and DII are facultative, and DIII is a “prolonged,” “deep” stasis compared to hatching delay and DIII in other killifishes, occurring just before environmentally cued hatching upon submersion in water (Wourms 1972c; Thompson and Ortí 2016; Thompson *et al.* 2017b). Pearlfish was suggested as a top candidate species for killifish models in the seminal work of developmental biologist John P. Wourms in 1967. They are small, prolific, and hardy, and spawn in sand (Wourms 1967), making them easily reared laboratory animals that are furthermore amenable to genetic manipulation like other killifishes (Aluru *et al.* 2015; Harel *et al.* 2015)*.* Pearlfish has also been an emergent system to study aging (Ruijter 1987), environmental influences on development (Ruijter *et al.* 1984), the role of prolactin in hatching control (Schoots *et al.* 1983; Ruijter and Creuwels 1988), resilience to perturbations in development with the ability to develop normally from diblastomeric eggs (Carter and Wourms 1993), and the transcriptional control of diapause and hatching (Thompson and Ortí 2016).

Here, we construct a chromosome-level genome assembly for the Rio Pearlfish, utilizing Hi-C contact maps, genome annotations, and gene expression analyses to characterize genomic evolution and hatching biology in this extremophile vertebrate.

## Materials and methods

### Genome sequencing and assembly

All animal work was approved by the Michigan State University Institutional Animal Care and Use Committee (PROTO202000108).

A total of 1.25 ng of template genomic DNA extracted from the liver of a single adult female *N. whitei* was loaded on a Chromium Genome Chip. Whole-genome sequencing libraries were prepared using 10× Genomics Chromium Genome Library & Gel Bead Kit v.2, Chromium Genome Chip Kit v.2, Chromium i7 Multiplex Kit, and Chromium controller according to the manufacturer’s instructions with one modification. Briefly, gDNA was combined with Master Mix, a library of Genome Gel Beads, and partitioning oil to create Gel Bead-in-Emulsions (GEMs) on a Chromium Genome Chip. The GEMs were isothermally amplified. Prior to Illumina library construction, the GEM amplification product was sheared on a Covaris E220 Focused Ultrasonicator to ∼350 bp and then converted to a sequencing library following the 10× standard operating procedure. A total of 679.43 million read pairs were sequenced on an Illumina HiSeqX sequencer, and a *de novo* assembly was constructed with Supernova 2.1.1 (Weisenfeld *et al.* 2018).

A Chicago library was prepared as described previously (Putnam *et al.* 2016). Briefly, ∼500 ng of HMW gDNA was reconstituted into chromatin *in vitro* and fixed with formaldehyde. Fixed chromatin was digested with DpnII, the 5′ overhangs were filled in with biotinylated nucleotides, and then free blunt ends were ligated. After ligation, crosslinks were reversed, and the DNA was purified from protein. Purified DNA was treated to remove biotin that was not internal to ligated fragments. The DNA was then sheared to ∼350 bp mean fragment size and sequencing libraries were generated using NEBNext Ultra enzymes and Illumina-compatible adapters. Biotin-containing fragments were isolated using streptavidin beads before PCR enrichment of each library. The libraries were sequenced on an Illumina HiSeqX to produce 242 million 2× 150 bp paired end reads.

A Dovetail Hi-C library was prepared in a similar manner as described previously (Lieberman-Aiden *et al.* 2009). For each library, chromatin was fixed in place with formaldehyde in the nucleus and then extracted. Fixed chromatin was digested with DpnII, the 5′ overhangs filled in with biotinylated nucleotides, and then free blunt ends were ligated. After ligation, crosslinks were reversed, and the DNA purified from protein. Purified DNA was treated to remove biotin that was not internal to ligated fragments. The DNA was then sheared to ∼350 bp mean fragment size and sequencing libraries were generated using NEBNext Ultra enzymes and Illumina-compatible adapters. Biotin-containing fragments were isolated using streptavidin beads before PCR enrichment of each library. The libraries were sequenced on an Illumina HiSeqX to produce 179 million 2 × 150 bp paired end reads.

The Supernova de novo assembly built from 10× Chromium data, Chicago library reads, and Dovetail Hi-C library reads were used as input data for assembly scaffolding with HiRise v1 (Putnam *et al.* 2016). An iterative analysis was conducted. First, Chicago library sequences were aligned to the draft input assembly using a modified SNAP read mapper (http://snap.cs.berkeley.edu). The separations of Chicago read pairs mapped within draft scaffolds were analyzed by HiRise v1 to produce a likelihood model for genomic distance between read pairs, and the model was used to identify and break putative misjoins, to score prospective joins, and make joins above a threshold. After aligning and scaffolding Chicago data, Dovetail Hi-C library sequences were aligned, and scaffolds were generated following the same approach.

### Genome annotation

The Rio Pearlfish genome was annotated with the NCBI Euakryotic genome annotation pipeline v9.0 (Thibaud-Nissen *et al.* 2016) and with MAKER 2.31 (Cantarel *et al.* 2008; Campbell *et al.* 2014; Bowman *et al.* 2017) using protein evidence from 15 fish species (Supplementary Table1) and transcriptome evidence from Rio Pearlfish DIII embryos and hatched larvae (Thompson and Ortí 2016). Genome assembly and annotation completeness (Supplementary Table2) were analyzed with BUSCO v5 (Simão *et al.* 2015) and CEGMA 2.4 (Parra *et al.* 2007) via the gVolante server (Nishimura *et al.* 2017, https://gvolante.riken.jp).

### Phylogenetics and orthology

To confirm species identification, we extracted and concatenated the barcoding marker genes *cox1* and *cytb* from our genome assembly, aligned them with orthologous sequences from all 3 described *Nematolebias* species (Costa *et al.* 2014) and inferred a phylogeny partitioned by codon and gene with RAxML (Stamatakis 2006, 2014) with the following parameters: -T 4 -N autoMRE -m GTRCAT -c 25 -p 12345 -f a -x 12345 –asc-corr lewis. We used Orthofinder v2.4.1 (Emms and Kelly 2015) to identify orthologous protein sequences between *N. whitei* and 35 other vertebrates genomes (Supplementary Table3) as well as protein sequences obtained from Cui *et al.* (2019), Hara *et al.* (2018), and the longest isoforms of other species available on NCBI RefSeq (last accessed 2021 September 22) downloaded with ortholog’s retrieve longest isoforms function (Drost *et al.* 2015). The output of Orthofinder (Supplementary Table4) was examined to identify Pearlfish-specific orthogroups. Genes in these orthogroups were used as queries in BLAST searches (e value cutoff of e−3) against Japanese medaka (HdrR strain, assembly ASM223467v1) protein sequences downloaded from Ensembl (last accessed 2022 January 17, Supplementary Table5). We performed a statistical overrepresentation test with a Fisher’s exact test and a false discovery rate correction on the Gene Ontologies (GOs) of these medaka genes using Panther v.16.0 (Mi *et al.* 2021) with the GO biological processes complete database (Supplementary Table6).

### Synteny and genome 3D structure

We examined conservation of synteny using genome assemblies and NCBI annotations for Rio Pearlfish, medaka (oryLat2, UCSC), and zebrafish (GCF_000002035.5_GRCz10, NCBI) as input for SynMap in the online CoGe database and toolkit (Lyons and Freeling 2008; last accessed October 14, 2021). Bwa v 0.7.17 (Li and Durbin 2009) was used to independently map Rio Pearlfish Hi-C read pairs to the genome assembly with the following parameters: bwa mem -A1 -B4 -E50 -L0, and HiCExplorer 3.6 (Ramírez *et al*. 2018) was used to construct a Hi-C matrix with the resulting bam files as follows: hicBuildMatrix –binSize 10000 –restrictionSequence GATC –danglingSequence GATC. The matrix was corrected via hicCorrectMatrix correct –filterThreshold -1.5 5. The matrix was binned depending on preferred resolution for viewing. Contact maps were visualized with hicPlotMatrix –log1p, and compared with contact maps of syntenic regions in medaka and zebrafish (Nakamura *et al.* 2021).

### Repeat content and repeat landscape

We constructed a species-specific repeat database with Repeat Modeler 2.0.1 (Smit and Hubley 2008). This library as well as vertebrate Repbase annotations (Jurka 2000) (downloaded 15 November 2017), and repeat libraries from platyfish (Schartl *et al.* 2013), coelacanth (Amemiya *et al.* 2013), bowfin (Thompson *et al.* 2021b), and spotted gar (Braasch *et al.* 2016) were combined to annotate repeat elements with RepeatMasker v4.0.5 (Smit *et al.* 2013). CalcDivergenceFromAlign.pl and createRepeatLandscape.pl in the RepeatMasker package were used to generate a repeat landscape. We graphically compared the repeat landscape of Rio Pearlfish to those described for other sequenced killifish species (Reichwald *et al.* 2015; Valenzano *et al.* 2015; Rhee *et al.* 2017; Cui *et al.* 2019) to identify similarities and difference in the magnitude and location of peaks at different Kimura distances in the histograms.

### Hatching enzyme gene expression

Aquatic vertebrates hatch by secreting choriolytic enzymes from hatching gland cells (HGCs) that break down the egg chorion (Yamagami 1988; Hong and Saint-Jeannet 2014). Teleost fishes underwent hatching enzyme gene duplications followed by divergence and functional divergence into the high choriolytic enzyme (*hce*) and low choriolytic enzyme (*lce*) genes (Yasumasu *et al.* 1992; Kawaguchi *et al.* 2006, 2010; Sano *et al.* 2014). We used BLAST to search the well-studied medaka hatching enzyme paralogs (*lce* and *hce*) against the annotated Pearlfish genome. We used the Pearlfish gene sequences from these BLAST hits as well as other metalloprotease gene sequences from medaka, *Austrofundulus, Kryptolebias*, and *Nothobranchius* (accession numbers in Supplementary Table7) to infer gene trees. The identified Pearlfish *lce* and *hce* genes are orthologous to those of other teleosts (data not shown). Pearlfish hatching enzyme orthologs were examined for transcript evidence from DIII embryos (Thompson and Ortí 2016) to identify active *lce* and *hce* gene expression in Pearlfish DIII.

We generated an antisense RNA probe for the Pearfish *lce.2* gene and performed whole-mount RNA *in situ* hybridization to identify hatching enzyme gene expression patterns as markers for the location of HGCs in Pearlfish. Total RNA was extracted from DIII Pearlfish embryos with a Qiagen RNeasy mini plus kit and reverse transcribed with a superscript IV VILO kit (ThermoFisher) according to the manufacturers’ instructions. The *lce* cDNA was amplified from the reverse transcribed template via PCR (Primers: Nwh_lce.1_1F: 5′-ATGGACCATAAAGCAAAAGTTTCTCTC-3′; Nwh_lce.1_792R: 5′-CTATTGCTTGTATTTTGAACACTTGT-3′; Nwh_lce.2_1F: 5′-ATGGACCATAAAGCAAAAGTTACTCTT-3′; Nwh_lce.2_825R: 5′-CTATTGCTTGTATTTTGAACAGTTGT-3′) and *lce.2* was inserted into a TOPO TA cloning kit vector (Invitrogen) according to the manufacturer’s instructions. Whole mount *lce.2* mRNA *in situ* hybridization on manually dechorionated DIII Rio Pearlfish embryos was performed following Deyts *et al.* (2005) with a 25 µg/ml proteinase K digestion treatment for 45 min (n = 3 embryos), 60 min (n = 3 embryos), and 90 min (n = 2 embryos).

## Results and discussion

### Genome sequencing and assembly

We report a high-quality, 1.2 Gb chromosome-level genome assembly of *N. whitei*. The Rio Pearlfish genome assembly consists of 24 chromosomal pseudomolecules represented by 24 superscaffolds that match the described karyotype (n = 24; Van Post, 1965). The assembly has an N50 of over 49.98 Mb and an L50 of 11 scaffolds (Table 1). BUSCO and CEGMA scores for different core gene databases indicate a high-quality assembly with an average of 94% complete BUSCOS and CEGs across all relevant databases (Table 1, Supplementary Table2).

**Table 1.** Rio Pearlfish genome assembly (NemWhi1) and annotation statistics.

**Genome assembly statistics**	
No. of scaffolds	18,999
No. of base pairs	1,218,332,341
N50	49,984,095
L50	11
N90	32,525,398
L90	22
No. of superscaffolds	24
No. of chromosomes (n)*^a^*	24
GC content	41.8%
Repeat content*^b^*	57.3%
BUSCO scores*^c^*: Vertebrata, Actinopterygii	96.9%, 95.5%
CEGMA scores*^c^*: CEG, CVG	99.19%, 99.57%
**Gene annotation statistics**	
**MAKER annotation**	
No. of protein-coding genes	26,016
BUSCO scores*^c^*: Vertebrata, Actinopterygii	91.4%, 86.2%
**NCBI RefSeq annotation***^d^*	
No. of protein-coding genes	21,341
BUSCO scores*^c^*: Vertebrata, Actinopterygii	97.4%, 96.5%
No. of genes in orthogroups*^e^*	21,176 (99.2%)
No. of species-specific orthogroups (genes)	17 (42)

a Van Post (1965).

b See Supplementary Table 10 for more information.

c See Supplementary Table 2 for more information.

d See Supplementary Table 8 for more information.

e See Supplementary Table 4 for more information.

### Genome annotation

The NCBI *N.* *whitei* Annotation Release 100.20210725 contains 23,038 genes, with 21,341 protein-coding genes, similar to other chromosome-level killifish genome assemblies from *N.* *furzeri* and *Kryptolebias marmoratus* (Reichwald *et al.* 2015; Valenzano *et al.* 2015; Kelley *et al.* 2016; Rhee *et al.* 2017) (Supplementary Table8). Minor differences in gene numbers among killifish species are likely due to annotation methods and minor species-specific gene losses or expansions. The number and content of annotated genes can be influenced by evidence used for annotation, differences in gene model prediction likelihoods, and postannotation filtering (Holt and Yandell 2011; Campbell *et al.* 2014). MAKER annotated 26,016 protein-coding genes, on par with the NCBI annotation. See Table 1 and Supplementary Tables 2 and 8 for Rio Pearlfish genome annotation statistics. Although our BUSCO analyses show fewer genes missed by the NCBI annotation (Supplementary Table2), our additional MAKER annotation provides additional, valid gene models missed by the NCBI pipeline. For example, MAKER annotates 28 vertebrate and 27 actinopterygian BUSCOs missed by the NCBI annotation pipeline (Supplementary Table9).

### Phylogenetics and orthology

Our orthofinder analysis illustrates the phylogenetic position of Rio Pearlfish among vertebrates (Fig. 1b) and identified 31,317 orthogroups across 36 vertebrate species with 99.2% of Rio Pearlfish genes within orthogroups (Table 1; Supplementary Table 4). We identified 7,287 orthogroups present across all species from sharks to human to Rio Pearlfish, highlighting the utility of the Rio Pearlfish genome to connect species with extreme developmental phenotypes to other vertebrates, including traditional vertebrate model species such as mouse, *Xenopus*, zebrafish, etc. We confirmed the identity of our genome specimen with barcoding and a molecular phylogeny of *cox1* and *cytb* with its position located within the *N. whitei* clade of *Nematolebias* killifishes (Fig. 1c). We found a total of 17 Pearlfish-specific orthogroups comprising a total of 42 protein sequences. For 39 of these, we established homology to a medaka gene class by BLAST (Supplementary Table5) and found an overrepresentation for GO terms related to glycolysis (Supplementary Table6). This may indicate an adaptive expansion of metabolic genes in this species as annual killifishes tolerate anoxia (Podrabsky *et al.* 2007, 2012; Wagner *et al.* 2018), severely depress metabolic rate during diapause (Podrabsky and Hand 1999), and drastically increase metabolic rate during fast maturation (Vrtílek *et al.* 2018) necessary for an annual life cycle. In a separate study, we have also found that genes involved in cell respiration, specifically oxidative phosphorylation, show higher ratios of nonsynonymous/synonymous codon changes in annual killifishes compared to their nonannual counterparts (Thompson *et al.* 2021a). Together, these observations points to potential positive selection on genes involved in cell respiration in annual killifishes.

### Synteny and genome 3D structure

**Fig. 2.**
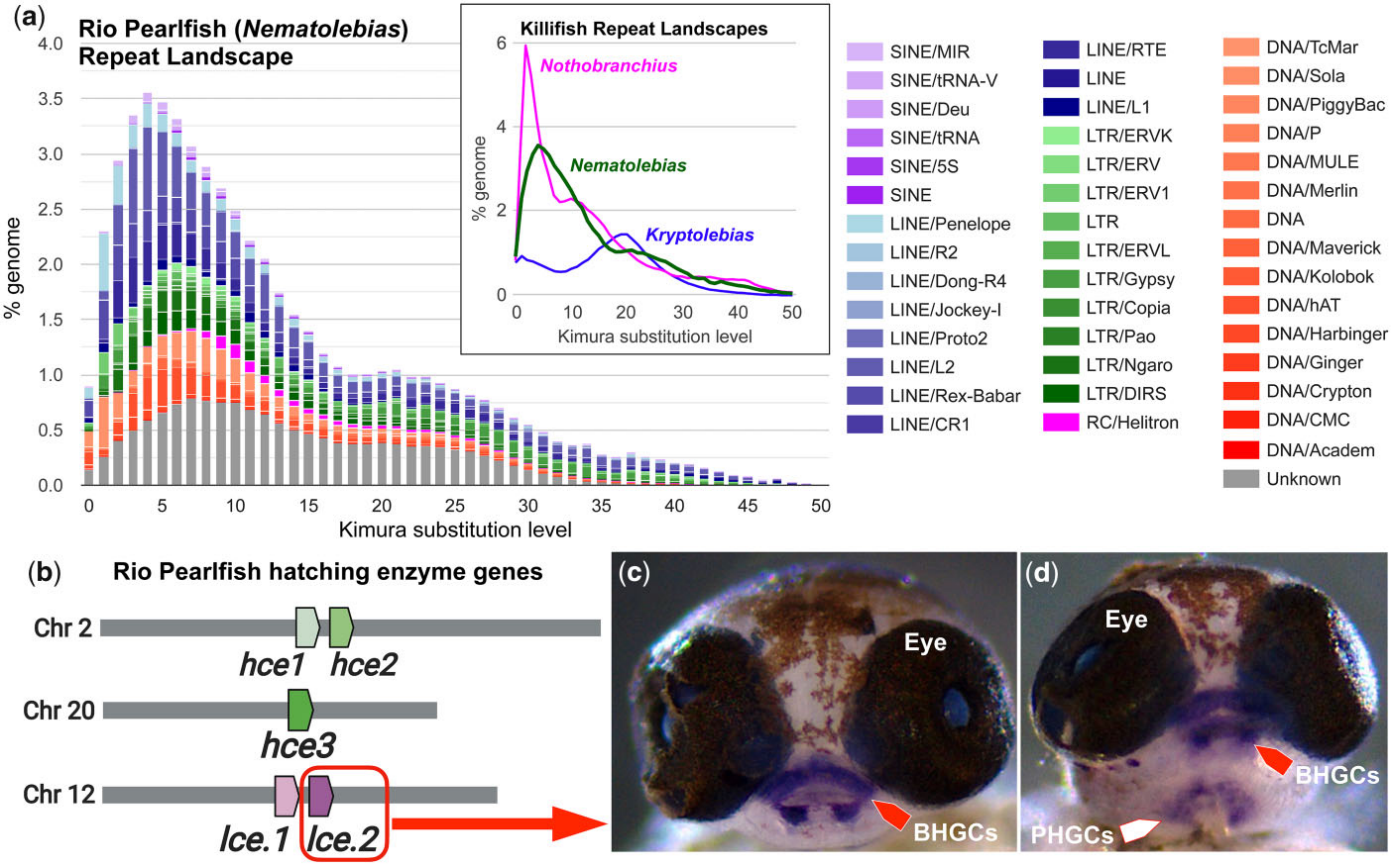
Rio Pearlfish repeat landscape, hatching enzyme genes, and hatching gland location. a) Repeat landscape of mobile genetic elements in Rio Pearlfish showing a high repeat content with 2 peaks at Kimura distance 4 and 21. Insert: Total TE landscape among killifishes with independent, recent expansions in the convergently annual *Nothobranchius* (Cui *et al.* 2019) and *Nematolebias* (this study) compared to the nonannual *Kryptolebias* (Choi *et al.* 2020). b) Locations of 5 hatching enzyme genes in the Rio Pearlfish genome expressed during DIII. c, d) Wholemount RNA *in situ* hybridization for *lce.2* in DIII Rio Pearlfish embryos marking hatching gland cells (HGCs) identified in the buccal (BHGCs, red arrows) and pharyngeal (PHGCs, white arrow) cavities.

Three-dimensional chromatin structure impacts gene regulation and can manifest as topologically associated domains (TADs) that could represent higher order gene regulatory regions conserved across evolution (Krefting *et al.* 2018). However, 3D genome structure has thus far remained uncharacterized in annual killifishes. To confirm the quality of the genome assembly and assess the utility of the chromatin conformation data to interrogate 3D genome structure and gene regulation, we constructed a Hi-C contact map showing higher contact frequency within the 24 pearlfish chromosomes (Fig. 1d) than between chromosomes. Using the genome sequence and gene annotations for Rio Pearlfish in synteny comparisons to another atherinomorph teleost, the medaka (separated by ∼85 million years), and the ostariophysian teleost zebrafish (separated by ∼224 million years), we reveal largely conserved synteny with these species (Fig. 2, e and f) across millions of years of teleost evolution (Hughes *et al.* 2018; Thompson *et al.* 2021a). We examined a TAD previously shown to be conserved from zebrafish to medaka (Nakamura *et al.* 2021) and found high frequency of contacts in the syntenic region between *rasa1a* and *mctp1a* in Rio Pearlfish liver tissue that resembles contact maps both in a medaka fibroblast cell line and zebrafish whole embryos (Fig. 1g). Hi-C analyses thus confirm the high-quality of our genome assembly as well as the strikingly conserved nature of 3D genome interactions across teleost evolution, developmental stages, and among cell and tissue types.

### Repeat content and transposable element landscape

Transposable elements (TEs) are hypothesized to generate novel genetic substrate for adaptations (Casacuberta and González 2013; Feiner 2016; Esnault *et al.* 2019). Some annual killifish species have expanded TE content compared to nonannual relatives (Cui *et al.* 2019), and the link between TEs, aging, and human diseases (Bravo *et al.* 2020) coupled with the rapid senescence of annual killifishes highlights the importance of examining the Pearlfish “mobilome.” We found that the Rio Pearlfish genome is highly repetitive with a repeat content of ∼57% (Fig. 2a and Table 1; Supplementary Table 10) which is substantially elevated compared to a nonannual member of the same South American family, *K.* *marmoratus*, with a repeat content of around ∼27% (Rhee *et al.* 2017; Choi *et al.* 2020). Similarly, African annual *Nothobranchius* killifishes have higher TE repeat content than their nonannual relatives (Cui *et al.* 2019). This pattern might be the result of adaptation to extreme environments as animals, fungi, and plants have co-opted TEs for environmental adaptations to harsh conditions (Casacuberta and González 2013; Esnault *et al.* 2019) and TEs may play roles in vertebrate adaptive radiations (Feiner 2016). Our findings further highlight the expanded repeat content in annual killifish genomes and the Pearlfish genome provides novel resources to study the role of mobile DNA in extremophiles.

### Hatching enzyme gene expression and hatching gland location

While hatching from the egg is a critical time point during animal development, little is known about its genetic regulation and the integration of environmental cues. Additionally, development of HGCs is dynamic among fishes (Inohaya *et al.* 1995; 1997) as they migrate and localize in different anatomical locations in different species (Korwin-Kossakowski 2012; Shimizu *et al.* 2014; Nagasawa *et al.* 2016). Pinpointing HGC location in seasonal killifishes is necessary for understanding the regulation of hatching in extreme environments.

Rio Pearlfish is a tractable model for studying hatching regulation since hatching is easily induced in this species by exposing DIII embryos to water (Thompson and Ortí 2016). Thus, we examined the hatching enzyme gene repertoire and HGC locations in Pearlfish. We identified five expressed hatching enzyme genes (Fig. 2b, 3 *hce* and 2 *lce* genes) upon mapping DIII mRNA reads from Thompson and Ortí (2016) to our reference genome assembly. We annotated *hce1* and *hce2* on chromosome 2 (corresponding to NCBI genes LOC119423801, LOC119423789), and *hce3* on chromosome 20 (LOC119426643) and the adjacent *lce.1* and *lce.2* genes (LOC119418488, LOC119418489) on chromosome 12 that are species-specific tandem duplicates (Fig. 2b) supported by transcript evidence (Thompson and Ortí 2016). Using whole mount RNA *in situ* hybridization for *lce.2* in DIII embryos, we identified HGC locations in the buccal and pharyngeal cavity in Rio Pearlfish (Fig. 2, c and d) similar to HGC localization in the related mummichog or Atlantic killifish (*Fundulus heteroclitus*) (Kawaguchi *et al.* 2005) and in medaka (Inohaya *et al.* 1995).

A pattern of expanded *hce* genes is also found in other Atherinomorph fishes like medaka. High choriolytic enzyme genes in this clade of teleosts have lost introns (Kawaguchi *et al.* 2010) and subfunctionalized postduplication with some *hce* genes performing better at higher or lower salinities in the euryhaline medaka *Oryzias javanicus* (Takehana *et al.* 2020) and the Atlantic killifish *F.* *heteroclitus* (Kawaguchi *et al.* 2013b)*.* Furthermore, the duplication of the *lce* gene in Rio Pearlfish is an example of convergent evolution within teleosts with another *lce* duplication in stickleback fishes (Kawaguchi *et al.* 2013a)*.* These findings underscore the commonality of hatching enzyme gene duplications in teleost fishes that provides a model system for studying convergent gene duplications and functional divergence by sub- and neofunctionalizations.

### Conclusions

Our chromosome-level, dually annotated genome assembly of the Rio Pearlfish provides a valuable comparative genomics resource strengthening the utility of killifishes for studying aging, suspended animation, and response to environmental stress. The Rio Pearlfish is an emerging “Extremo” Eco-Evo-Devo research organism, and this reference genome will be a substrate for future functional genetic and multiomic approaches exploring how organisms integrate developmental and environmental cues to adapt to extreme environmental conditions in a changing world.

## Data availability

The genome sequence, annotation, and sequence read data are available on NCBI under accession GCA_014905685.2 and Bioproject PRJNA560526. The genome assembly and annotation has also been integrated to the University of California Santa Cruz Genome Browser (https://hgdownload.soe.ucsc.edu/hubs/fish/index.html). The MAKER genome annotation is available on github (https://github.com/AndrewWT/NematolebiasGenomics).

Supplemental material is available at *G3* online.

## Supplementary Material

jkac045_Supplementary_Data
